# Transcriptomic investigations of polymyxins and colistin/sulbactam combination against carbapenem-resistant *Acinetobacter baumannii*

**DOI:** 10.1016/j.csbj.2024.05.043

**Published:** 2024-05-31

**Authors:** Xingchen Bian, Mengyao Li, Xiaofen Liu, Yan Zhu, Jian Li, Phillip J. Bergen, Wanzhen Li, Xin Li, Meiqing Feng, Jing Zhang

**Affiliations:** a Institute of Antibiotics, Huashan Hospital, Fudan University, Shanghai, China; b Key Laboratory of Clinical Pharmacology of Antibiotics, Shanghai, China; c National Health Commission & National Clinical Research Center for Aging and Medicine, Huashan Hospital, Fudan University, Shanghai, China; d Department of biological medicines & Shanghai Engineering Research Center of Immunotherapeutics, School of Pharmacy, Fudan University, Shanghai, China; e Clinical Pharmacology Research Center, Huashan Hospital, Fudan University, Shanghai, China; f Department of Critical Care Medicine, The Second Clinical Medical College, Jinan University (Shenzhen People's Hospital), Shenzhen, China; g Integrated Chinese and Western Medicine Postdoctoral Research Station, Jinan University, Guangzhou, China; h Tianjin Institute of Industrial Biotechnology, Chinese Academy of Sciences, China; i Biomedicine Discovery Institute and Department of Microbiology, Monash University, Melbourne, Australia

**Keywords:** Carbapenem-resistant *Acinetobacter baumannii*, Polymyxins, Sulbactam, Combination, Transcriptomics

## Abstract

Carbapenem-resistant *Acinetobacter baumannii* (CRAB) is a Priority 1 (Critical) pathogen urgently requiring new antibiotics. Polymyxins are a last-line option against CRAB-associated infections. This transcriptomic study utilized a CRAB strain to investigate mechanisms of bacterial killing with polymyxin B, colistin, colistin B, and colistin/sulbactam combination therapy. After 4 h of 2 mg/L polymyxin monotherapy, all polymyxins exhibited common transcriptomic responses which primarily involved disruption to amino acid and fatty acid metabolism. Of the three monotherapies, polymyxin B induced the greatest number of differentially expressed genes (DEGs), including for genes involved with fatty acid metabolism. Gene disturbances with colistin and colistin B were highly similar (89 % common genes for colistin B), though effects on gene expression were generally lower (0–1.5-fold in most cases) with colistin B. Colistin alone (2 mg/L) or combined with sulbactam (64 mg/L) resulted in rapid membrane disruption as early as 1 h. Transcriptomic analysis of this combination revealed that the effects were driven by colistin, which included disturbances in fatty acid synthesis and catabolism, and inhibition of nutrient uptake. Combination therapy produced substantially higher fold changes in 72 % of DEGs shared with monotherapy, leading to substantially greater reductions in fatty acid biosynthesis and increases in biofilm, cell wall, and phospholipid synthesis. This indicates synergistic bacterial killing with the colistin/sulbactam combination results from a systematic increase in perturbation of many genes associated with bacterial metabolism. These mechanistic insights enhance our understanding of bacterial responses to polymyxin mono- and combination therapy and will assist to optimize polymyxin use in patients.

## Highlights

• All polymyxins exhibited common modes of action which primarily involved disruption to amino acid and fatty acid metabolism.
• Colistin/sulbactam therapy produced substantially higher fold changes in 72 % of DEGs shared with monotherapy, indicating a systematic synergism.
• These mechanistic insights enhance our understanding of bacterial responses to polymyxin mono- and combination therapy.

## Graphical Abstract

**Figure:**
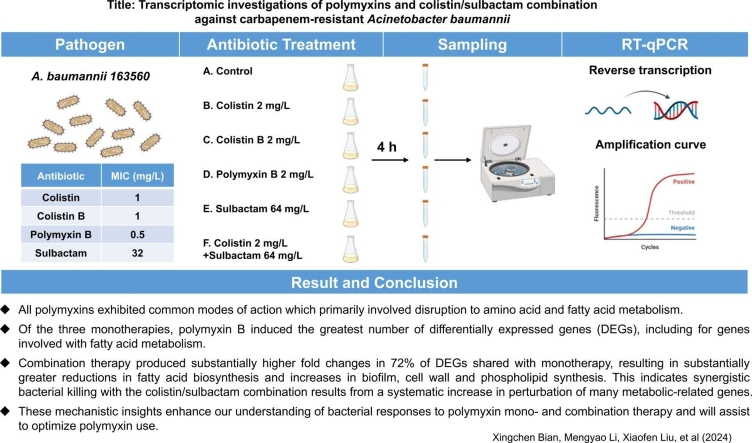

## 1 Introduction

In 2017, carbapenem-resistant *Acinetobacter baumannii* (CRAB) was highlighted by the World Health Organization (WHO) as one of three Priority 1 (Critical) pathogens for which new antibiotics were urgently needed [1]. Over the last 15 years, the prevalence of carbapenem resistance in clinical *A. baumannii* in China has increased from ∼40 % to ∼75 % [2]. Given these bacteria are often resistant to not only the carbapenems but also β-lactams, aminoglycosides, and fluoroquinolones [3], [4], therapeutic options are limited. While the polymyxins (colistin and polymyxin B) retain substantial activity against many problematic Gram-negative organisms and are considered a last-line treatment option against CRAB, polymyxin resistance is increasingly being reported [5]. Given dose escalation is precluded because of nephrotoxicity which occurs in up to 60 % of patients receiving intravenous polymyxins [6], [7], polymyxin combination therapy is generally recommended [8]. A recent systematic review and meta-analysis concluded that colistin combined with sulbactam was safer and more efficacious than colistin monotherapy against CRAB-associated infections [9], and we also recently reported synergistic bacterial killing against three clinical isolates of CRAB with this combination in an *in vitro* pharmacokinetic/pharmacodynamic model [10]. However, the mechanism(s) underpinning synergy have not been fully investigated.

Colistin (aka polymyxin E) and polymyxin B became available clinically approximately 60 years ago. Colistin is administered parenterally in the form of the inactive prodrug, colistin methanesulphonate (CMS), and polymyxin B directly as its active (sulphate) form [11]. In China, a parenterally-administered colistin sulphate formulation has also recently become available [12]. Both colistin and polymyxin B consist of groups of lipopeptides, with the two main components in commercial products being colistin A and B (for colistin) and polymyxin B1 and B2 (for polymyxin B) [11]. Colistin A and B differ solely at their *N*-terminal fatty acid where colistin A contains 6-methyloctanoic acid and colistin B 6-methyheptanoic acid (Fig. S1) [13]. Both colistin and polymyxin B have almost identical minimum inhibitory concentrations (MICs) [14], [15]. A formulation of colistin B (aka polymyxin E2), which has been shown in animal models to have similar antibacterial activity to colistin but lower nephrotoxicity and neurotoxicity, is currently undergoing Phase I clinical trials (CTR20211397).

The polymyxins bind to lipopolysaccharide (LPS) before inserting into the outer membrane of Gram-negative bacteria, resulting in expansion of the outer membrane monolayer [16]. While membrane disruption is believed to be the primary mode of action of the polymyxins, additional events such as inhibition of NADH-quinone oxidoreductase and generation of hydroxyl radicals have additionally been proposed to contribute to bacterial killing [17], [18]. While it is known that there are commonalities in the mechanism(s) of action of colistin and polymyxin B due to their structural similarities [19], few studies have investigated whether the structural differences associated with the various polymyxins confer alternative mechanisms of action. While traditional molecular biological techniques have primarily focused on examining a single mechanism of action, the development of multi-omics technology has allowed the biological processes underpinning drug action to be examined more thoroughly, including combination drug therapy [20], [21]. Accordingly, the primary aim of this study was to examine the similarities and differences in the transcriptomic responses of *A. baumannii* to treatments with polymyxin B, colistin, and, for the first time, colistin B. Additionally, the study investigated how the combination of colistin and sulbactam leads to improved bacterial killing of *A. baumannii*. This study will enhance our understanding of the modes of action of polymyxins and their combinations with sulbactam.

## 2 Results

### 2.1 Differentially expressed genes

**Fig. 1:**
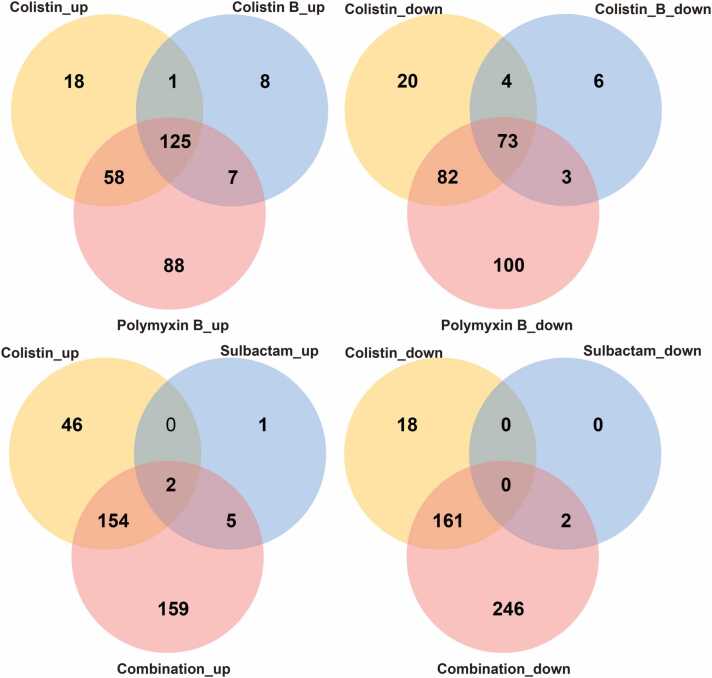
The numbers of common and unique up- or down-regulated DEGs in *A. baumannii* 163560 following 4 h of treatment with polymyxin or sulbactam monotherapy (2 mg/L of colistin, colistin B, polymyxin B, or 64 mg/L sulbactam) or the combination of 2 mg/L colistin plus 64 mg/L sulbactam.

The heatmap shown in Fig. S2 illustrates the effective clustering of the samples from both the control and each treatment group. The number of up- and down-regulated DEGs is shown in Fig. 1. Across the three polymyxin monotherapies, 125 and 73 genes were commonly up-regulated and down-regulated, respectively. Polymyxin B resulted in substantially more specific DEGs than either colistin or colistin B, with 88 up-regulated and 100 down-regulated. The total number of DEGs with sulbactam monotherapy was minimal (8 [0, 1, 2 and 5] up-regulated and 2 [0, 0, 0 and 2] down-regulated). However, the total number of DEGs observed with the colistin/sulbactam combination was almost twice that of colistin alone (729 *vs.* 381 total DEGs, respectively). The number of common DEGs between the colistin and combination groups was substantially greater (156 [154 and 2] up-regulated and 161 down-regulated), while the largest number of specific DEGs was observed with the combination (159 up-regulated and 246 down-regulated).

### 2.2 KEGG enrichment analysis and COG functional classification

The KEGG pathways with FDR < 0.05 are presented in Table S1. The small number of DEGs observed with sulbactam monotherapy were associated with glycolysis/gluconeogenesis. Only the ABC transporter pathway was enriched with all polymyxin monotherapies and combination therapy. The ABC transporter pathway is involved in various biological functions which is annotated as membrane transport here. These observations are in accordance with the known targets of colistin and sulbactam. In total, 5 pathways were enriched with colistin monotherapy and 3 with colistin B, polymyxin B, sulbactam monotherapy, and combination therapy.

**Fig. 2:**
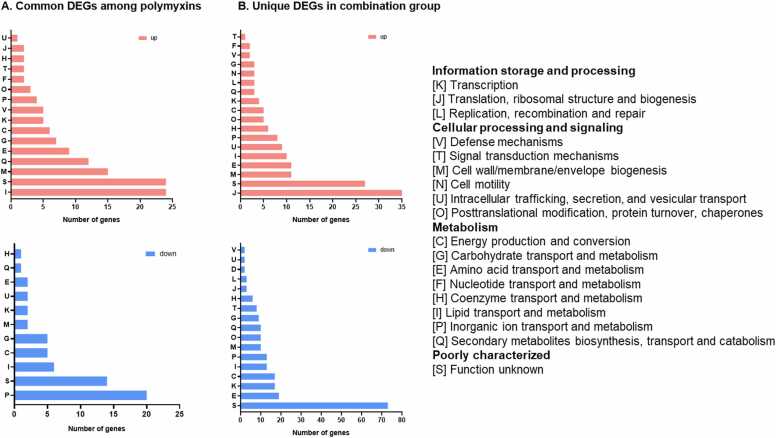
COG category of (A) common up- and down-regulated DEGs with 2 mg/L polymyxin monotherapy (colistin, colistin B, or polymyxin B), and (B) unique DEGs with combination treatment (2 mg/L colistin plus 64 mg/L sulbactam). Data shown is following 4 h of antibiotic treatment.

The whole genome of *A. baumannii* 163560 was annotated into 21 COG classifications. Genes within the whole genome were primarily engaged in transcription (K), inorganic ion transport and metabolism (P), energy production (C), cell wall/membrane/envelope biogenesis (M), and lipid transport and metabolism (I). Relevant changes in the number of genes with polymyxin monotherapy and combination treatment are shown in Fig. 2. With polymyxin monotherapy, commonly up-regulated genes were notably associated with cell wall/membrane/envelope biogenesis and lipid transport and metabolism, while the most prevalent down-regulated genes were involved in inorganic ion transport and metabolism (Fig. 2A). With combination treatment, the largest number of up-regulated genes were associated with translation, ribosomal structure, and biogenesis (Fig. 2B). These nutrients transport and membrane biosynthesis alterations were in alignment with the enrichment observed in ABC transporter (membrane transport) as shown in Table S1.

### 2.3 DEGs in response to polymyxin monotherapy

**Fig. 3:**
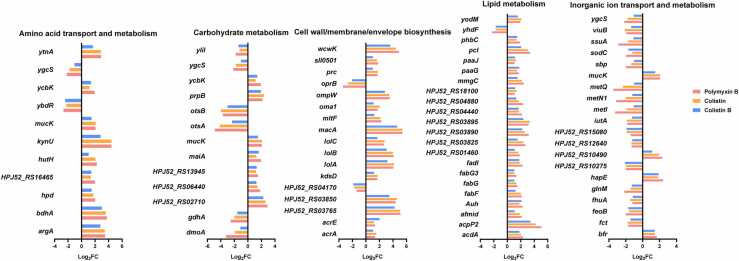
Fold changes of common DEGs associated with highly perturbed COG categories following 4 h of treatment with 2 mg/L polymyxin monotherapy (colistin, colistin B, or polymyxin B).

The fold changes of common DEGs associated with highly perturbed COG categories with each polymyxin monotherapy are shown in Fig. 3 and Table S2. Of the DEGs shared by all three polymyxins, fatty acid synthesis genes *fabG, fabF, acpP2*, HPJ52_RS03825, HPJ52_RS03890, as well as fatty acid catabolism genes *fadI, mmgC, acdA, paaG, paaJ*, and HPJ52_RS04880, were all up-regulated 2- to 32-fold; *paaG* and *paaJ* are also associated with phenylacetic acid catabolism in aromatic amino acid metabolism [22], [23]. Of genes associated with cell wall/membrane/envelope biosynthesis, the lipoprotein transport-related genes *lolA, lolB*, and *lolC* were up-regulated 2- to 18-fold. The *oprB* gene encoding a glucose-selective porin that promotes the diffusion of glucose to the outer membrane [24] was down-regulated 4- to 11-fold, while the macrolide export protein-encoding gene *macA* was up-regulated 24- to 43-fold. For genes involved in amino acid metabolism, *ybdR* (encoding threonine dehydrogenase) was down-regulated 4- to 7-fold, while *ytnA* (encoding γ-aminobutyric acid permease), *hutH* (encoding histidine ammonia lyase), and *argA* (encoding N-acetylglutamate synthase) were up-regulated 6- to 12-fold. N-acetylglutamate synthase is involved in the first step of bacterial arginine synthesis by catalyzing the formation of N-acetylglutamate from glutamate and acetyl-CoA [25]. Glutamine ABC transporter-encoding gene *glnM* was down-regulated 2- to 5-fold. The directional change (i.e., up- or down-regulated) of all DEGs common to each polymyxin monotherapy was consistent, with polymyxin B producing the most substantial fold changes for most genes (Fig. 3).

**Fig. 4:**
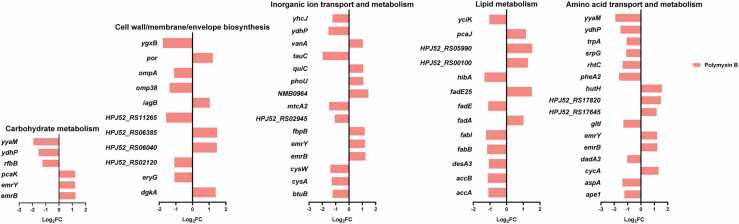
Fold changes of unique DEGs associated with highly perturbed COG categories following 4 h of treatment with 2 mg/L polymyxin B monotherapy.

Only 14 specific genes were associated with colistin B monotherapy, with fold changes near the cutoff (log_2_FC = 1). While 7 of 14 were annotated as hypothetical proteins, other genes were involved in amino acid and fatty acid metabolism. As described above, substantially more specific DEGs (88 up-regulated and 100 down-regulated) were induced by polymyxin B compared to either colistin or colistin B (Fig. 1). In addition to the common DEGs associated with fatty acid metabolism, the fatty acid catabolism gene *fadA*, which catalyzes the final step of fatty acid β-oxidation from ketoacyl-CoA to acetyl-CoA and fatty acyl-CoA with two less carbons (https://www.uniprot.org/uniprot/P21151), was up-regulated 2-fold with polymyxin B monotherapy (Fig. 4). The newly generated fatty acyl-CoA can participate in the β-oxidation cycle and continuously produce acetyl-CoA. Fatty acid synthesis genes *accA, accB, desA3, fabB*, and *fabI* were all down-regulated 2- to 3-fold. *accA* and *accB* encode acetyl-CoA carboxylase, while *fabB* encodes β-ketoacyl synthase and *fabI* enoyl-[acyl-carrier-protein] reductase. These gene alterations indicate reduced fatty acid synthesis with polymyxin B monotherapy. In the cell wall/membrane/envelope biosynthesis pathway, *ompA* (which encodes an outer membrane protein that is an essential virulence factor of *A. baumannii* [26]), was down-regulated ∼2-fold (Fig. 4). In the amino acid transport pathway, the genes encoding tryptophan synthase (*trpA*) and cysteine synthase (*srpG*) were each down-regulated 2- to 3-fold, suggesting a reduction of tryptophan and cysteine synthesis (Fig. 4).

### 2.4 DEGs in response to colistin monotherapy and colistin/sulbactam combination therapy

**Fig. 5:**
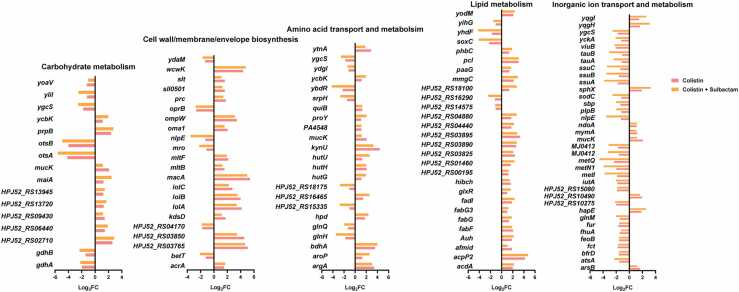
Fold changes of common DEGs associated with highly perturbed COG categories following 4 h of treatment with 2 mg/L colistin monotherapy or 2 mg/L colistin combined with 64 mg/L sulbactam.

When the DEGs associated with colistin monotherapy and colistin/sulbactam combination therapy were compared, both treatments caused up-regulation of genes involved in fatty acid metabolism (*fabG, fabF, acpP2, fadI, mmgC, acdA*, and *paaG*) and lipoprotein transport (*lolA, lolB*, and *lolC*). Conversely, numerous genes associated with inorganic ion transport and metabolism including amino acid transport and metabolism, metal ion transport, and phosphate, sulfate, and sulfonate transport were down-regulated (Fig. 5). For example, the L-glutamine ABC transporter periplasmic binding protein/ATP binding subunit *glnH/glnQ*, and sulfate ABC transporter protein-encoding gene *sbp*, were down-regulated 2- to 11-fold and 2- to 4-fold, respectively. In glucose metabolism, *gdhA* and *gdhB*, which encode glutamate dehydrogenase which catalyzes the dehydrogenation of glutamate to α-ketoglutarate in the tricarboxylic acid cycle [27], [28], were down-regulated 4- to 5-fold and 2- to 5-fold, respectively. Additionally, *otsA* and *otsB* which encode, respectively, trehalose 6-phosphate synthase and phosphatase, and *glnH* and *glnQ*, jointly responsible for glutamine transport, were all down-regulated 2- to 48-fold (Fig. 5).

**Fig. 6:**
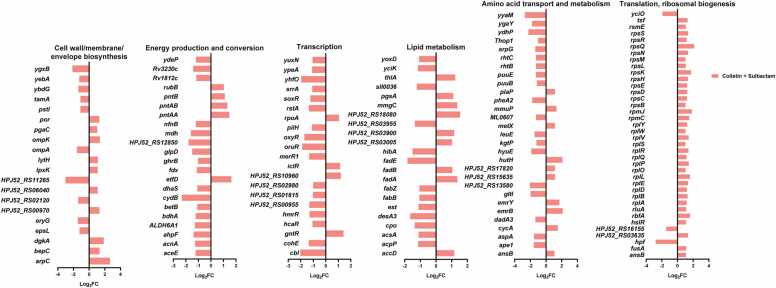
Fold changes of unique DEGs associated with highly perturbed COG categories following 4 h of treatment with 2 mg/L colistin combined with 64 mg/L sulbactam.

Among the specific DEGs associated with combination therapy were genes responsible for ribosomal biosynthesis and translation, including genes encoding 30 s and 50 s ribosomal proteins (all up-regulated) and transcriptional regulators (all down-regulated) (Fig. 6). In addition to up-regulation of the common fatty acid metabolism genes, combination therapy additionally caused the up-regulation of fatty acid catabolism genes (*fadA* and *fadB*) and down-regulation of biosynthesis genes (*acpP*, *fabB*, *fabZ* and *desA3*). In the cell wall/membrane/envelope biosynthesis pathway, genes involved in phospholipid synthesis (*dgkA*) and *N*-acetyl-D-glucosamine biosynthesis (*pgaC*) were up-regulated 3.6- and 2.0-fold, respectively (Fig. 6); *pgaC* is also associated with biofilm formation [29]. In the amino acid transport and metabolism pathway, *gltI* (involved in the glutamate/arginine ABC transporter complex) and *aspA* (which catalyzes the deamination of aspartate to fumarate) were significantly down-regulated (3.9- and 2.9-fold, respectively) (Fig. 6).

As observed in the RNA-Seq analyses, *fabF, fabG, lolB*, and *lolC* were expressed at significantly increased levels relative to the untreated control with colistin and polymyxin B monotherapy and the colistin/sulbactam combination, but not with colistin B monotherapy (Fig. S3).

### 2.5 Cell morphology and membrane integrity with colistin and sulbactam treatment

**Fig. 7:**
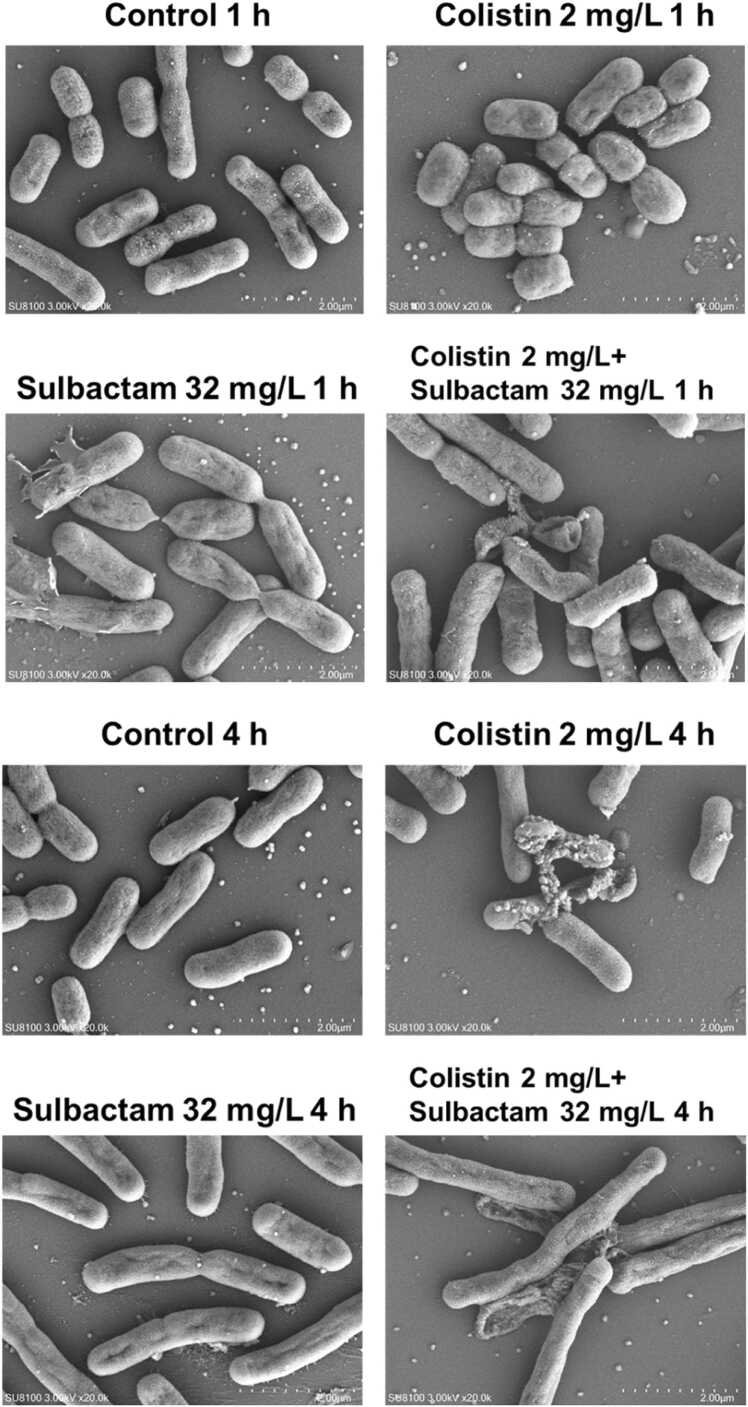
Scanning electron microscopy showing cell morphology of *A. baumannii* 163560 at 1 h and 4 h in the absence of antibiotic treatment (Control) and following exposure to 2 mg/L colistin and 32 mg/L sulbactam as mono- or combination therapy.

**Fig. 8:**
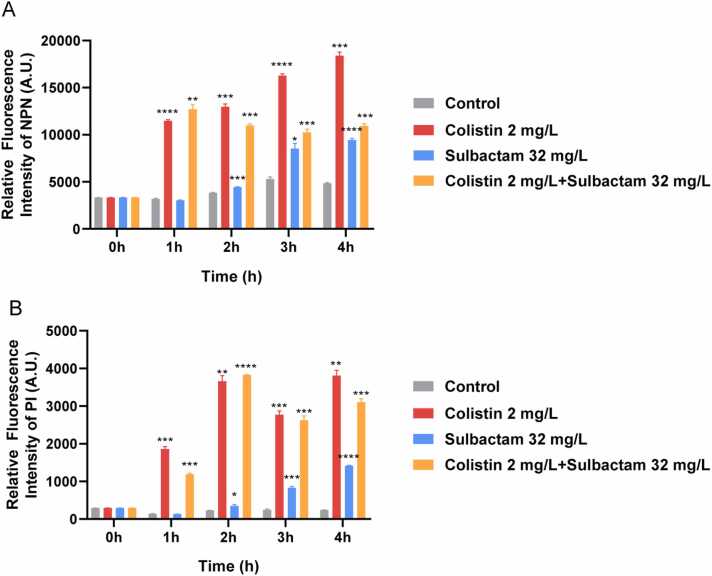
The fluorescence intensity across 4 h of (A) N-phenyl-1-naphthylamine (NPN) and (B) propidium iodide (PI) following no antibiotic treatment (Control) and treatment with colistin and sulbactam mono- and combination therapy. * 0.05 < p < 0.01, * *0.01 < p < 0.001, * ** 0.001 < p < 0.0001, * ** *p < 0.0001.

The outer membrane of Gram-negative bacteria plays a major role in safeguarding these organisms from detrimental external factors by excluding toxic molecules such as antibiotics and providing an additional stabilizing layer around the cell [30]. SEM images and alterations in the permeability of the inner and outer membranes in the absence and presence of antibiotic treatment are shown in Fig. 7, Fig. 8, respectively. In the control group, rod-shaped bacteria in various stages of cell division were observed at both 1 and 4 h. However, cell length was reduced at 1 h with colistin monotherapy, while it was elongated at 4 h with sulbactam monotherapy or combination therapy. Cell rupture or morphological depression was apparent with colistin or sulbactam monotherapy and combination therapy (Fig. 7). Disruptions of cell integrity were observed with colistin monotherapy and the colistin/sulbactam combination as early as 1 h, while the effects of sulbactam monotherapy on membrane integrity were observed from 2 h (Fig. 8). When the outer membrane is damaged, NPN binds to exposed hydrophobic phospholipid tails and fluoresces. When both the inner and outer membranes are disrupted, PI penetrates the cell and binds to DNA, also fluorescing. The heightened fluorescence we observed with colistin or sulbactam treatments suggests considerable damage to the outer membrane (Fig. 8). Interestingly, fluorescence with 2 mg/L colistin monotherapy was greater than with the combination at all time points, likely due to a greater number of live bacteria with colistin monotherapy (Fig. 8A). However, the PI fluorescence signal across all time points with 2 mg/L colistin monotherapy was similar to that of combination regimens (Fig. 8B).

## 3 Discussion

While several studies have utilized transcriptomics to investigate bacterial responses to either colistin [31], [32], [33] or polymyxin B [31], [32], [34], this technique has never been utilized to investigate responses to colistin B or a polymyxin/sulbactam combination. While it is known that the structural similarities between colistin and polymyxin B elicit broadly similar responses in Gram-negative bacteria, it is less well understood whether the structural differences between the various polymyxins likewise confer different responses and therefore different mechanism(s) of action. The present study is the first to investigate the transcriptomic responses of colistin B and to specifically compare response differences between the various polymyxins. We additionally investigated the transcriptomic alterations related to mechanism(s) underpinning the synergy observed with colistin/sulbactam combinations [10].

In agreement with previous studies examining colistin or polymyxin B [31], [32], [33], [34], functional analysis of the DEGs associated with polymyxin monotherapy, including colistin B, revealed generally similar effects on bacteria metabolism. These included down-regulation of fatty acid biosynthesis and up-regulation of lipoprotein transport, indicative of membrane remodeling in response to membrane damage. Interestingly, we previously reported in MDR *Klebsiella pneumoniae* that *fabG* (responsible for fatty acid biosynthesis) was down-regulated at 1 h in response to 2 mg/L polymyxin B monotherapy and a polymyxin B/caspofungin combination, and upregulated at 4 h only with combination therapy, indicating time-dependent regulation of fatty acid metabolism [34]. We have also shown that the fatty acid biosynthesis genes *fabDBGZI* were down-regulated in at least 3 of 5 polymyxin-susceptible *A. baumannii* strains at 15 or 60 min in response to polymyxin B or colistin treatment [31]. However, in the present study we observed up-regulation of *fabG* at 4 h with both colistin and combination therapy. Similar up-regulation of genes encoding the Lol lipoprotein transport system in *A. baumannii* have been reported in response to polymyxin B and/or colistin treatment, as was observed in the present study [31], [32], [33]. In contrast, prior research reported significant upregulation of stress response genes, including *feoABC* and *ssuAC* (fold changes ranging from 11.3 to 90.5), in *Pseudomonas aeruginosa* exposed to 4 mg/L polymyxin B for 1 h [35]*.* However, our study found that *feoB* and *ssuABC* were down-regulated 3–4 and 2–15-fold at 4 h, respectively, in response to each polymyxin monotherapy or the colistin/sulbactam combination. Given previous reports of time-dependent effects on gene expression in response to polymyxin therapy [31], [33], [34], such inverse regulation of stress response genes may reflect time-dependent bacterial responses upon polymyxin exposure.

For common DEGs among the various polymyxin monotherapies, polymyxin B was associated with the most significant disturbances. Several specific DEGs were also identified with polymyxin B monotherapy. Notable among these was the ∼2-fold down-regulation of *ompA*, which encodes the outer membrane protein OmpA. In *A. baumannii*, OmpA is essential for the regulation of adhesion, invasiveness, biofilm formation, and maintenance of cell envelope integrity [26], [36]. OmpA may also couple with efflux pumps to extrude antibiotics from the periplasm, leading to antibiotic resistance [37]. Recently, OmpA was shown to contribute to colistin resistance in *A. baumannii* via an isogenic OmpA mutant which exhibited a reduction in cell wall integrity and 20-fold greater sensitivity to colistin than the wild-type strain [38]. The expression level of *ompA* in *A. baumannii* has been proposed as a rapid diagnostic indicator for drug-resistant *A. baumannii* given it is highly correlated with susceptibility to a wide variety of antibiotics, including colistin [39]. The reduced expression of *ompA* with polymyxin B therapy (and colistin/sulbactam combination) suggests polymyxin B likely reduces antibiotic efflux and decreases bacterial adhesion *in vivo* to facilitate bacterial clearance. The increased disturbance of common DEGs by polymyxin B, combined with its induction of substantially more specific DEGs, may explain the increased susceptibility of *A. baumannii* 163560 to polymyxin B (MIC, 0.5 mg/L) compared to colistin and colistin B (MICs of 1 mg/L). Surprisingly, colistin B caused only minimal gene disturbances despite an identical MIC to that of colistin. Nevertheless, while some differences in the degree of disruption of DEGs and induction of specific DEGs was discernable between each polymyxin monotherapy, the fold-changes observed with polymyxin B were not substantially greater than those observed with colistin and, consequently, no differences in mechanism of action were discernable.

With both colistin monotherapy and the colistin/sulbactam combination, the common DEGs were enriched in cell envelope biosynthesis and inorganic ion transport. Most genes encoding ABC transporter proteins were down-regulated, indicating nutrient-deficiency which may inhibit bacterial growth. However, not all nutrients benefit bacterial growth when antibiotics are present. For example, exogenous glutamine can facilitate killing of MDR bacteria, including *A. baumannii*, by increasing antibiotic uptake [40]. In our study, down-regulation of *glnH*, *glnQ*, and *glnM* (all associated with glutamine transport) was observed, suggesting a reduction in glutamine and antibiotic uptake with colistin mono- and combination therapy. Similarly, trehalose can support bacterial survival in adverse situations, such as peroxidation, and help maintain bacterial integrity [41], [42]. With colistin mono- and combination therapy, the expressions of *otsA* and *otsB* (which encode trehalose 6-phosphate synthase and trehalose phosphatase, respectively) was substantially reduced (by 17.7/48.0-fold for *otsA* and 16.0/30.2-fold for *otsB*), suggesting major disruptions to this self-protection barrier which would help facilitate bacterial killing.

Compared with colistin alone, the colistin/sulbactam combination induced more significant disturbances in a wide range of common DEGs, indicating that the combination produced synergism through the systematic regulation of bacterial metabolism. As both polymyxins and sulbactam target the bacterial cell envelope, alterations of the cell envelope are predicted to be closely associated with the observed synergism when used in combination [40], [41]. Following their initial binding to LPS in the bacterial outer membrane, the polymyxins displace divalent cations (Ca^2+^ and Mg^2+^) and the hydrophobic N-terminal fatty-acyl polymyxin tail of the polymyxin inserts into the membrane. As a consequence, the packing of adjacent lipid A molecules is weakened, thereby inducing the expansion of the outer membrane monolayer and ultimately cell lysis [19]. Sulbactam is a β-lactamase inhibitor typically combined with cefoperazone or ampicillin. However, unlike other β-lactamase inhibitors, it also has intrinsic activity against *A. baumannii* due to an ability to acetylate and thereby inhibit transpeptidase activity of penicillin-binding proteins (PBPs, mainly PBP1 and PBP3), hindering the synthesis of peptidoglycan and thus the bacterial cell wall [43], [44]. It’s worth noting that several hypothetical proteins were consistently upregulated across the three polymyxin monotherapies and the combination therapy. The afmid gene was associated with lipid metabolism, while oma1 and HPJ52_RS03850 are involved in cell wall/membrane/envelope biosynthesis. These trends suggest bacterial adaptation with the drug treatment. These hypothetical proteins could also be part of alternative metabolic pathways that were activated in response to the antibiotics.

Of DEGs unique to combination therapy, *pgaC* and *dgkA*, which are involved in cell wall and phospholipid synthesis, respectively, were upregulated. The *pgaABCD* locus of *A. baumannii* encodes proteins that synthesize cell-associated poly-β-(1−6)-N-acetylglucosamine (PNAG), which is crucial for biofilm formation [45], [46]. Interestingly, we have previously reported that the *pgaABCD* genes were up-regulated in response to colistin or polymyxin B monotherapy in all 5 *A. baumannii* strains examined in a pan-transcriptomic study [31]. Additionally, these genes were up-regulated in response to colistin monotherapy and a colistin/doripenem combination in a single *A. baumannii* reference strain and its colistin-resistant, LPS-deficient *lpxA* mutant strain in our PK/PD study [33]. Activation of *pgaC* with combination therapy suggests the triggering of biofilm formation to assist bacterial survival. Collectively, upregulation of *pgaC* and *dgkA* suggests that, in response to the colistin/sulbactam combination, bacteria initiate membrane repair and reduce the charge-based interaction of the positively-charged colistin with the bacterial outer membrane via a repulsive effect from the positively-charged PNAG [33]. Another intriguing change was that almost all the DEGs that encoded ribosomal protein were upregulated. Further studies are required to illustrate whether the upregulation of genes encoding ribosomal proteins contribute to their synergism. These transcriptomic data demonstrating the benefit of polymyxin/sulbactam combinations such as presented here will provide reference for future in-depth investigations of synergistic mechanisms.

In summary, this transcriptomic study demonstrated that despite their structural differences, polymyxin B, colistin, and colistin B all lead to similar transcriptomic responses in *A. baumannii*, resulting in the upregulation of genes involved in fatty acid synthesis and catabolism, cell wall/membrane/envelope biosynthesis, and lipoprotein transport. Genes involved with inorganic ion transport and metabolism were downregulated. Synergy between colistin and sulbactam induced more significant disturbances in a wide range of common DEGs, with the mechanism of synergy involving systematic regulation of bacterial metabolism that primarily targeted the cell envelope. These findings highlight the critical role of the cell envelope in the bacterial response to antibiotic treatment. The mechanistic synergism between colistin and sulbactam suggests a promising approach for enhancing antibacterial efficacy. This study provides valuable insights that could inform the development of more effective combination therapies against *A. baumannii*.

## 4 Materials and methods

### 4.1 Bacterial strain, antibiotics, media and susceptibility testing

MDR (including carbapenem-resistant) clinical isolate *A. baumannii* 163560, taken from a patient with a bloodstream infection, was used in this study [10]. The genome assembly of *A. baumannii* 163560 is available with accession number ASM1456030v1. We have previously determined the sequence type of this strain to be ST 191, belonging to the most epidemiological clonal type 208 (CC208) [47]. The genetic distance of *A. baumannii* 163560 was relatively close to strains AB5075_UW, AYE, and ACICU, which have been widely used in published papers[47]. The combination of colistin and sulbactam was chosen for mechanistic investigation based on our previous study which reported synergistic activity against *A. baumannii, especially A. baumannii* 163560, using checkerboard and *in vitro* static and dynamic PK/PD studies [10]. The above reasons made *A. baumannii* 163560 a representative and optimal strain for this study. The antibiotics used were polymyxin B (77.53 %, USP, R046V0), colistin (79.3 %, Meilun Biotech, N0620A), colistin B (86.3 %, CTTQ, 20140529), and sulbactam sodium (99.8 %, Meilun Biotech, O0210AS). Minimum inhibitory concentrations (MICs) were determined in duplicate using broth microdilution as per CLSI guidelines [48]. The MICs were 0.5 mg/L for polymyxin B, 1 mg/L for colistin and colistin B, and 32 mg/L for sulbactam. Prior to all experiments, strains were subcultured onto Luria-Bertani (LB) agar (Sangon, A507003) from frozen stock (−80 °C), and incubated at 37 °C for 18–24 h. Cation-adjusted Mueller Hinton Broth (CAMHB; Ca^2+^ at 20–25 mg/L, Mg^2+^ at 10–12.5 mg/L; Becton Dickinson, Sparks, MD) was employed for all bacterial cultures.

### 4.2 Transcriptomic sample preparation and sequencing

Bacterial colonies were inoculated into 10 mL of CAMHB and incubated overnight (37 °C, 150 rpm). Overnight cultures were diluted 1:100 with fresh CAMHB and grown to an optical density at 600 nm (OD_600_) of 0.5 (∼10^8^ CFU/mL) before drug treatment. Sterile drug solutions (in Milli-Q water) were then added to give final concentrations of 2 mg/L for polymyxin B, colistin, and colistin B, and 64 mg/L for sulbactam (all monotherapy), or 2 mg/L colistin and 64 mg/L sulbactam (combination therapy). Antibiotic concentrations were determined based on the bacterial killing observed in preliminary time-kill experiments (Fig. S4), and were chosen to ensure sufficient bacterial counts (∼10^8^ CFU) for RNA extraction. An untreated bacterial culture acted as the control. All experiments were conducted in triplicate with bacterial samples collected after 4 h of antibiotic treatment. RNA extraction and sequencing was performed by Personalbio (Shanghai) using the Illumina Novaseq 6000 platform (paired-end, 2 ×150 bp).

### 4.3 Data processing and analyzing

Data filtering and quality assessment were conducted for raw data by Personalbio (Shanghai). The filtered reads were then mapped to the reference genome [47] using bowtie2 [49], and the read counts for each gene obtained as the expression profile. To compare gene expression between each gene and sample, read counts were normalized to fragments per kilobase per million mapped fragments (FPKM). The R package pheatmap was applied to construct a heatmap using z-score [50]. Read counts of each gene were normalized by R package DESeq and described as basemean values. The p-values were adjusted using the Benjamini-Hochberg method [51] to control the false discovery rate (FDR). Differentially expressed genes (DEGs) were identified using a combination of fold change ≥ 2 and FDR ≤ 0.05. Venn diagrams were drawn by the online tool jvenn (http://jvenn.toulouse.inra.fr/app/example.html) [52].

The web-based server KAAS (KEGG Automatic Annotation Server: http://www.genome.jp/kegg/kaas/) was used for functional annotation [53]. Enrichment analysis was performed using the R package clusterProfiler [54]. KEGG enrichment analysis, the GeneRatio (i.e., ratio of the number of DEGs enriched in one pathway to the number of all annotated DEGs), FDR value, and the number of genes enriched in one pathway, were used to determine whether the pathway was significantly perturbed. The Clusters of Orthologous Groups of proteins (COGs) database [55] was applied to classify proteins using eggnog mapper (http://eggnog-mapper.embl.de/) [56].

### 4.4 Real-time quantitative PCR

Given fatty acid biosynthesis was significantly disturbed by antibiotic treatment, the target genes chosen were fatty acid biosynthesis genes (*fabF* and *fabG*) and lipoprotein transport-associated genes (*lolB* and *lolC*). The *A. baumannii* housekeeping gene *gyrB* was selected as the internal reference [33]. Primer sequences were designed as shown in Table S3. For RNA extraction, 3 mL of each bacterial culture was collected after 4 h of no treatment (the control) or antibiotic treatment, and RNA extracted using a TaKaRa MiniBEST Universal RNA Extraction Kit (Takara) according to the manufacturer’s instructions. PrimeScript™ RT reagent Kit with gDNA Eraser (Takara) and FastStart Universal SYBR Green Master (Roche) were used for reverse transcription and real-time quantitative PCR (RT-qPCR), respectively.

cDNA synthesis, amplification, and quantification were then undertaken with primer concentrations of 0.3 μM on a ViiA7 fluorescence quantitative PCR instrument programed with the following: 50 °C/2 min, 95 °C/10 min, 40 cycles of 95 °C/15 s, followed by + 60 °C/1 min. The delta-delta Ct method (also known as the 2^-∆∆Ct^ method) was adopted to calculate the relative fold changes of gene expressions, where Ct stands for the cycle threshold of each sample, ∆Ct is the difference in Ct values for the targeted gene and the internal reference gene, and ∆∆Ct is the difference between the ∆Ct values of the treated sample and the control sample [57].

### 4.5 Scanning electron microscopy (SEM) and membrane integrity studies

For both the SEM and membrane integrity studies, bacterial cultures were prepared as per the transcriptomic study prior to the addition of either no antibiotic (control group), or 2 mg/L colistin, 32 mg/L sulbactam, or 2 mg/L colistin plus 32 mg/L sulbactam. For the SEM study undertaken to observe cell morphology, 1 mL of each bacterial solution was removed following 1 h and 4 h of antibiotic treatment and centrifuged at 3000 rpm for 5 min. The supernatant was discarded, cell pellets resuspend in 200 μL of phosphate-buffered saline (PBS, pH 7.4), and 10 μL pipetted onto polyethylenimine-coated coverslips. The coverslips were then immersed for another 2 h in 2.5 % glutaraldehyde in PBS before thrice being rinsed for 10 min in PBS. Dehydration was then performed using increasing concentrations of ethanol in water (10 %, 30 %, 50 %, 70 %, 90 % and 100 %) for 10 min per step [58]. The coverslips were dried and gold coated. Cells were imaged with a HITACHI SU8100-emission SEM at a voltage of 3 kV.

Examination of the integrity of the inner and outer membranes at 1, 2, 3 and 4 h following no treatment or antibiotic treatment was undertaken as previously described [59]. Briefly, bacterial pellets were rinsed twice and resuspended in 1 mL of PBS. To examine inner membrane integrity, 8 μL of 500 μM N-phenyl-1-naphthylamine (NPN, 98 %, Merk, 104043) dissolved in ethanol was added to 1 mL of bacteria suspension. To examine outer membrane integrity, 5 μL of 1 mM propidium iodide (PI, ≥94 %, Merk, P4170) dissolved in sterile water was added to 1 mL of bacteria suspension. After mixing, 100 μL was transferred to a 96 microwell plate and incubated in the dark for 30 min. The excitation and emission wavelengths were set to 350 nm and 420 nm for NPN, and 535 nm and 617 nm for PI [59]. The fluorescence intensity was obtained at each timepoint. Membrane integrity studies were performed in triplicate.

## Ethics approval and consent to participate

Sample collection was approved by the Huashan Institutional Review Board. All strains used in this study belong to The Culture Collection of Institute of Antibiotics, Huashan Hospital. The Informed Consent Form was waived by the Huashan Institutional Review Board if strains from The Culture Collection were used for further study. Personal privacy is not involved in this study.

## Funding

This research was supported by 10.13039/501100001809National Natural Science Foundation of China (82173896) and Shanghai Municipal Science and Technology Major Project (HS2021SHZX001).

## CRediT authorship contribution statement

**Xingchen Bian:** Writing – review & editing, Writing – original draft, Visualization, Methodology, Formal analysis, Data curation, Conceptualization. **Mengyao Li:** Visualization, Software, Methodology, Data curation. **Xiaofen Liu:** Validation, Supervision, Project administration, Methodology, Conceptualization. **Yan Zhu:** Software, Methodology, Investigation, Data curation. **Jian Li:** Supervision, Project administration, Methodology, Conceptualization. **Phillip J. Bergen:** Writing – review & editing, Methodology, Investigation. **Wanzhen Li:** Methodology, Investigation, Data curation. **Xin Li:** Methodology, Formal analysis, Data curation. **Meiqing Feng:** Supervision, Project administration, Investigation. **Jing Zhang:** Writing – review & editing, Supervision, Project administration, Funding acquisition, Conceptualization.

## Declaration of Competing Interest

None.

## Data Availability

The raw and processed data of transcriptomic sequencing can be accessed by the accession number GSE218219 in GEO database.
