# Supplementary material for: Transcriptomic investigations of polymyxins and colistin/sulbactam combination against carbapenem-resistant Acinetobacter baumannii
Source: Comput Struct Biotechnol J. 2024 May 31;23:2595–605. doi: 10.1016/j.csbj.2024.05.043 (PMC11245955; doi:10.1016/j.csbj.2024.05.043)
Supplement: Supplementary file 1 — Supplementary material. [file mmc1.docx]

**Transcriptomic investigations of polymyxins and colistin/sulbactam combination against carbapenem-resistant *Acinetobacter baumannii***

**Xingchen Bian^a,b,c,d,e^, Mengyao Li^f,g^, Xiaofen Liu^a,b,c^, Yan Zhu^h^, Jian Li^i^, Phillip J. Bergen^i^, Wanzhen Li^a,b,c^, Xin Li^a,b,c^, Meiqing Feng^d^, Jing Zhang^a,b,c,e*^**

Institute of Antibiotics, Huashan Hospital, Fudan University, Shanghai, China^a^;

Key Laboratory of Clinical Pharmacology of Antibiotics, Shanghai, China^b^;

National Health Commission & National Clinical Research Center for Aging and Medicine, Huashan Hospital, Fudan University, Shanghai, China^c^;

Department of biological medicines & Shanghai Engineering Research Center of Immunotherapeutics, School of Pharmacy, Fudan University, Shanghai, China^d^;

Clinical Pharmacology Research Center, Huashan Hospital, Fudan University, Shanghai, China^e^;

Department of Critical Care Medicine, The Second Clinical Medical College, Jinan University (Shenzhen People's Hospital), Shenzhen, China^f^;

Integrated Chinese and Western Medicine Postdoctoral Research Station, Jinan University, Guangzhou, China^g^;

Tianjin Institute of Industrial Biotechnology, Chinese Academy of Sciences, China^h^.

Biomedicine Discovery Institute and Department of Microbiology, Monash University, Melbourne, Australia^i^.

^*^Corresponding author: Jing Zhang, phone number: +86 021-52888193, postal address: 12 Middle Wulumuqi Road, Jing’an District, Shanghai, China, e-mail address: zhangj61@fudan.edu.cn


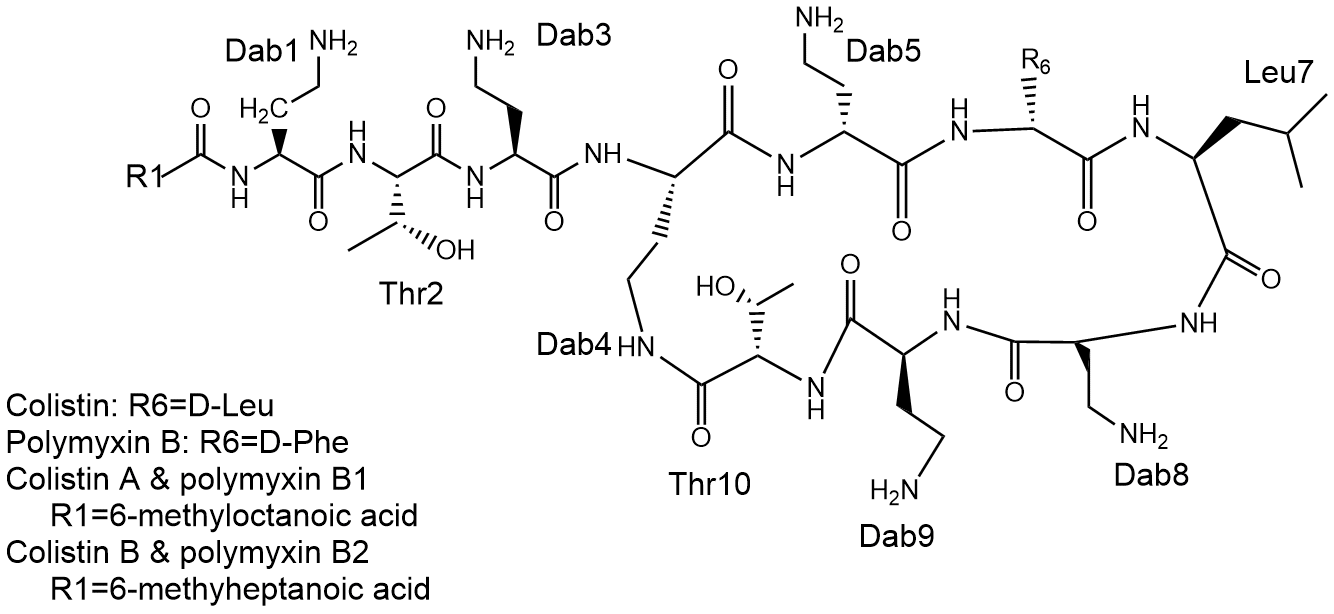


**Fig. S1** Chemical structures of Polymyxins. Dab, 2,4-diaminobutyric acid, Thr, Threonine, Leu, Leucine.


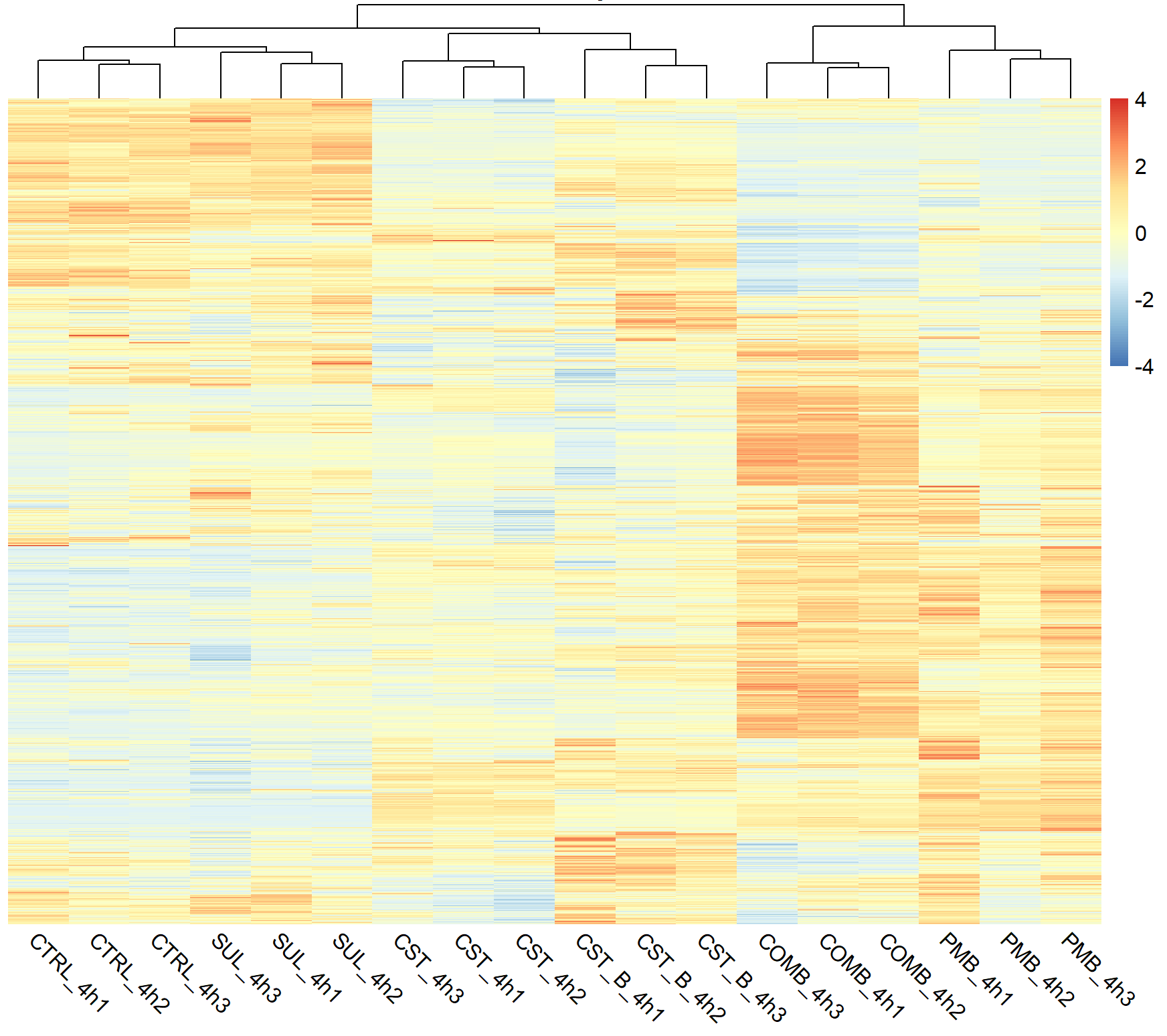


**Fig. S2.** Heatmap of gene expression in *A. baumannii* 163560 in the control and treatment groups at 4 h. CRTL, control; CST, 2 mg/L colistin; CST_B, 2 mg/L colistin B; PMB, 2 mg/L polymyxin B; SUL, 64 mg/L sulbactam; COMB, 2 mg/L colistin plus 64 mg/L sulbactam.

**Fig. S3**. Gene expression levels at 4 h in *A. baumannii* 163560 from RT-qPCR and RNA-seq. Abbreviations: CST, 2 mg/L colistin; CST_B, 2 mg/L colistin B; PMB, 2 mg/L polymyxin B; COMB, 2 mg/L colistin plus 64 mg/L sulbactam. Each sample in the RT-qPCR had three technical replicates. Only DEGs identified by RNA-seq in each group are shown.

**Fig. S4.** Time-killing curves of *A. baumannii* with the treatment of polymyxins, sulbactam and their combination. Abbreviations: CST, colistin; CST_B, colistin B; PMB, polymyxin B; COMB, colistin plus sulbactam.

**Table S3.** Primer sequences used in RT-qPCR

| **Primer** | **Sequence (5’ to 3’)** |
| --- | --- |
| *fabF*-F | GGTGCCTACCACAATGGCTA |
| *fabF*-R | CCTGATGCTATGCGGTCTGT |
| *fabG*-F | ACTACGCTCGCCACTTCATC |
| *fabG*-R | GCCTTAGCACTCGAATTGGC |
| *lolB*-F | AGTTGGCTCACACGATTTGC |
| *lolB*-R | GGCTGGCAAGCACCAATTAC |
| *lolC*-F | GCATTGGTTTCGATGGTCGG |
| *lolC*-R | TGAAACTGTCGCTTGCGGTA |
| *gyrB*-F | CGAGGGTGACTCAGCGGGTG |
| *gyrB*-R | GCGCACGCTCAACGTTCAGG |
